# Intestinal melatonin levels and gut microbiota homeostasis are independent of the pineal gland in pigs

**DOI:** 10.3389/fmicb.2024.1352586

**Published:** 2024-03-26

**Authors:** Jiaming Zheng, Yewen Zhou, Di Zhang, Kezhe Ma, Yuneng Gong, Xuan Luo, Jiali Liu, Sheng Cui

**Affiliations:** ^1^College of Veterinary Medicine, Yangzhou University, Yangzhou, China; ^2^Jiangsu Co-Innovation Center for Prevention and Control of Important Animal Infectious Diseases and Zoonoses, Yangzhou University, Yangzhou, China; ^3^State Key Laboratory of Animal Biotech Breeding, College of Biological Sciences, China Agricultural University, Beijing, China; ^4^Institute of Reproduction and Metabolism, Yangzhou University, Yangzhou, China

**Keywords:** pig, pinealectomy, melatonin, intestine, gut microbiota

## Abstract

**Introduction:**

Melatonin (MEL) is a crucial neuroendocrine hormone primarily produced by the pineal gland. Pinealectomy (PINX) has been performed on an endogenous MEL deficiency model to investigate the functions of pineal MEL and its relationship with various diseases. However, the effect of PINX on the gastrointestinal tract (GIT) MEL levels and gut microbiome in pigs has not been previously reported.

**Methods:**

By using a newly established pig PINX model, we detected the levels of MEL in the GIT by liquid chromatography–tandem mass spectrometry. In addition, we examined the effects of PINX on the expression of MEL synthesis enzymes, intestinal histomorphology, and the intestinal barrier. Furthermore, 16S rRNA sequencing was performed to analyze the colonic microbiome.

**Results:**

PINX reduced serum MEL levels but did not affect GIT MEL levels. Conversely, MEL supplementation increased MEL levels in the GIT and intestinal contents. Neither PINX nor MEL supplementation had any effect on weight gain, organ coefficient, serum biochemical indexes, or MEL synthetase arylalkylamine N-acetyltransferase (AANAT) expression in the duodenum, ileum, and colon. Furthermore, no significant differences were observed in the intestinal morphology or intestinal mucosal barrier function due to the treatments. Additionally, 16S rRNA sequencing revealed that PINX had no significant impact on the composition of the intestinal microbiota. Nevertheless, MEL supplementation decreased the abundance of Fibrobacterota and increased the abundance of Actinobacteriota, Desulfobacterota, and Chloroflexi.

**Conclusion:**

We demonstrated that synthesis of MEL in the GIT is independent of the pineal gland. PINX had no influence on intestinal MEL level and microbiota composition in pigs, while exogenous MEL alters the structure of the gut microbiota.

## 1 Introduction

Melatonin (MEL), a crucial neurohormone derived from tryptophan, is mainly synthesized by the pineal gland during the dark phase of the light/dark cycles (Claustrat and Leston, 2015; Yin et al., 2020). Synthesis of extra-pineal MEL was reported in the retina (Wiechmann and Sherry, 2013), skin (Rusanova et al., 2019), Harderian gland (Santillo et al., 2020), and, importantly, the gastrointestinal tract (GIT) (Bubenik, 2002; Pan et al., 2021). MEL is synthesized in the enterochromaffin cells of the GIT (Huether, 1993; Kvetnoy et al., 2002), and it is known that the GIT contains approximately 400 times more MEL than the pineal gland (Bubenik, 2002; Chen et al., 2011). Therefore, MEL is not only important in regulating the circadian rhythm but is also involved in regulating multiple gastrointestinal functions, such as intestinal motility (De Filippis et al., 2008), barrier permeability (Sommansson et al., 2013; Swanson et al., 2015), energy expenditure (Prezotto et al., 2014; Xu et al., 2022), and bicarbonate secretion (Sommansson et al., 2013, 2014). In addition, it was reported that MEL deficiency is closely associated with the development of intestinal diseases (Gong et al., 2022; Xia et al., 2022). Furthermore, MEL improved sleep deprivation-induced intestinal mucosal injury by altering the composition of the gut microbiota (Gao et al., 2019, 2021). More importantly, clinical studies show that sleep disturbances increase disease activity in patients with gastrointestinal disorders (Sochal et al., 2020), indicating an association between MEL deficiency and gastrointestinal diseases.

Gut microbiota is crucial for the homeostatic maintenance of gastrointestinal function. Studies have demonstrated that MEL could influence the swarming and motility of *Enterobacter aerogenes* (Paulose et al., 2016). Moreover, in high-fat diet-fed mice, MEL has been proven to prevent obesity and obesity-related disorders by modulating the diversity and composition of gut microbiota, including *Firmicutes, Bacteroides*, and *Akkermansia* (Xu P. et al., 2017). Although it is clear from these studies that administration of exogenous MEL affects the gut microbiota, it is unclear whether deficiency of endogenous MEL affects intestinal barrier integrity and gut microbiota dysbiosis. Previous research has reported that some MEL in the intestine may originate from the pineal gland through circulation, especially at nighttime (Huether et al., 1998). On the contrary, other research studies have shown that MEL is locally synthesized in the intestine and is independent of the pineal gland since PINX has no influence on its concentration (Huether, 1993; Bubenik and Brown, 1997; Kvetnoy et al., 2002). Thus, it is necessary and meaningful to understand the effect of PINX and MEL supplementation on intestinal MEL levels and gut microbiota.

PINX was performed on an endogenous MEL deficiency model to evaluate the regulation of circadian rhythms in the pineal gland (de Farias et al., 2015; Tchekalarova et al., 2020), immunity (Sahin et al., 2018; Luo et al., 2020), Alzheimer's disease (Tzoneva et al., 2021), and inflammation and oxidative stress (Ballur et al., 2022). However, most of these studies were performed on rodents (Al Gburi et al., 2022; Demir et al., 2022), so it is difficult to use the findings of these studies to explain various MEL deficiency disorders in humans because nocturnal rodents and diurnal humans have different circadian rhythms in terms of behavior and physiology (Cheung et al., 2005; Slawik et al., 2016). Pigs are highly similar to humans in terms of physiological anatomy, nutritional metabolism, cardiovascular system structure, and immune response, making them one of the ideal experimental animal models for the study of human diseases (Walters et al., 2017; Pabst, 2020). Previously, PINX was found to promote small intestine crypt cell proliferation in rats (Callaghan, 1991). However, to date, no experimental studies have been conducted on pigs to assess the impact of PINX on intestinal function and gut microbiota.

In this study, we used a newly established pig PINX model to evaluate the effects of pineal and exogenous MEL on growth performance, biochemical parameters, GIT MEL levels, intestinal mucosal barrier function, and subsequent gut microbiome composition. These findings demonstrate that MEL synthesis in the GIT is independent of the pineal gland in pigs. Exogenous MEL, not pineal MEL, affects the gut microbiota.

## 2 Materials and methods

### 2.1 Reagents and antibodies

Antibodies against arylalkylamine N-acetyltransferase (AANAT) (ab108508) and GAPDH (ab15580) were purchased from Abcam (Cambridge, UK). ZO-1 (TA5145) and claudin-1 (T56872) were purchased from Abmart (Shanghai, China). MEL and formic acid were purchased from Macklin Biochemical Co., Ltd. (Shanghai, China). Methanol was purchased from Sigma (St. Louis, MO, USA).

### 2.2 Animals

A total of 18 male Bama pigs (5–6 months old), with an initial body weight of approximately 18–20 kg, were obtained from Beijing Farm Animal Research Center, Beijing, China. They were housed in the SPF lab of Yangzhou University, with a temperature of 24 ± 2°C and humidity of 60 ± 5%, under a 12-h light–dark cycle. The animals were fed with standard diet at 8:00 a.m., 12:00 p.m., and 18:00 p.m. based on their weight, with *ad libitum* access to drinking water. Experiments were approved by the Animal Ethics Association of Yangzhou University (approval ID: SYXK (Su) 2022-0044).

### 2.3 Experimental design and treatment with MEL

Eighteen pigs were divided on average into three groups: sham–pinealectomy group (Control), pinealectomy group (PINX), and pinealectomy + melatonin supplementation group (PINX+MEL). A total of 12 pigs underwent stereotaxic surgery with the removal of the pineal gland, and other six pigs received the same procedure without the removal of the pineal gland. Two weeks after the PINX operation, PINX pigs were randomly divided into two groups: the PINX group receiving equal amounts of ethanol and the PINX+MEL group receiving 10 mg/kg of body weight MEL daily at 18:00 pm in drinking water for 4 months, according to the earlier literature (Agil et al., 2011). The MEL was dissolved in ethanol and further diluted in 2,000 mL water. The body weights were monitored every 2 weeks.

### 2.4 Surgery procedure

The surgical procedure of PINX on pigs has recently been established in our laboratory by applying a parieto-occipital approach (unpublished data) (Dempsey et al., 1982; Egermann et al., 2011). First, a near circular-shaped incision was made above the parietal bone by using a circular drill and an angled rongeur. The bone flap was delicately removed to expose the dura mater, which was then opened to expose the bilateral parietal lobes. The parieto-occipital lobes were retracted bilaterally by using a brain retractor. Dissection was performed down to the pineal recess, and the pineal gland was then observed medial to the internal cerebral veins. The pineal gland was grasped with delicate forceps and removed in one motion. Successful removal of the pineal gland was confirmed by autopsy and a decrease in serum MEL levels.

### 2.5 Collection of samples

Four months after surgery, the animals were euthanized by the carotid artery bleeding under anesthesia with 2% pentobarbital sodium (0.5 mL/kg) at 20:00 p.m. After laparotomy, the brain, heart, liver, spleen, lung, kidney, and testis were excised and weighted. The middle part of the GIT from the stomach to the rectum and samples of the digesta from six intestinal segments were collected as previously described (Zhang et al., 2022). After washing with phosphate-buffered saline, one part of the GIT was fixed with 4% paraformaldehyde and the other was frozen in liquid nitrogen. The relative organ weight was calculated according to the formula (Xia et al., 2022): relative organ weight: organ weight/body weight × 100.

### 2.6 Serum lipid indexes

Blood samples were collected from the anterior vena cava after 12 h of fasting, and serum was separated by centrifuging at 3,500 rpm according to previous literature (Xia et al., 2022). An automatic biochemistry analyzer (C16000; Abbott Architect, IL, USA) was used to test alanine aminotransferase (ALT), aspartate aminotransferase (AST), total cholesterol (TC), triglyceride (TG), high-density lipoprotein (HDL-C), and low-density lipoprotein (LDL-C) levels.

### 2.7 Hematoxylin and eosin (H&E) staining

The duodenum and ileum were dehydrated in gradient alcohol series, cleared in xylene, and then processed into paraffin sections. Paraffin blocks of 7 mm were cut and stained with H&E. The sections were analyzed by using a light microscope (IX71; Olympus, Tokyo, Japan). Villus length and crypt depth were measured according to previous literature (Ren et al., 2018).

### 2.8 MEL analysis

A volume of 300 μL of serum was suspended in 1 mL ethyl acetate, vortexed fully for 3 min, and ultrasonicated for 1 min to extract the MEL. For intestine and intestinal contents, 0.1 g of samples was ground to a fine powder with liquid nitrogen and suspended in ethyl acetate. After centrifuging at 12,000 g for 15 min under 4°C, the supernatants were collected and dried using nitrogen gas. Furthermore, the samples were redissolved in 150 μL mobile phase (0.1% formic acid–methanol = 65: 35, v/v), vortexed, and centrifuged. After filtration with a 0.22-um filter, the supernatants were transferred to an autosampler vial. LC-MS/MS was performed according to the method described by Magliocco et al. (2021). Briefly, samples were analyzed by an ExionLC high-performance liquid chromatography (HPLC) system coupled to a 6500 QTRAP mass spectrometer (ABI Sciex, Foster City, CA, USA). An Agilent ZORBAX Eclipse Plus C18 column (4.6 mm x 100 mm, 3.5 um particle size) (Agilent Technologies, Santa Clara, CA, USA) was used with a temperature of 40°C. The mobile phase was water with 0.1% formic acid (A) and methanol with 0.1% formic acid (B). The elution programs are detailed in Table 1. The ion spray voltage was 5.5 kV in the ESI mode. Quantitation was performed in the positive ionization mode by multiple reaction monitoring at *m/z* 233.1/174.0.

**Table 1:** Elution programs of MEL analysis.

**Time (min)**	**Flow rate (mL/min)**	**%A**	**%B**
Initial	0.6	60	40
0.8	0.6	60	40
2.0	0.6	20	80
4.0	0.6	20	80
4.5	0.6	60	40
5.5	0.6	60	40

### 2.9 Western blotting

Western blotting was performed as described in previous literature (Zhang et al., 2018). First, the sample was electrophoresed using 12% SDS-PAGE gel and transferred to the PVDF membrane. After blocking with 5% skim milk, the membrane was incubated with AANAT, ZO-1, claudin-1, and GAPDH at 4°C overnight. After washing with TBST, they were incubated with the secondary antibody for 2 h at room temperature. The protein bands were detected with electrochemiluminescence (Vazyme Biotech, Nanjing, China). Band intensities were quantified by using ImageJ software.

### 2.10 Gut microbiota analysis

Fresh colonic contents (~200 mg of each sample) were collected from each animal at the same time. Bacterial genomic DNA was extracted from samples using the Power Fecal^®^ DNA Isolation Kit (MoBio Carlsbad, CA USA). The V3–V4 regions of the 16S rRNA gene were amplified using primers 341F and 806R (5′-CCTAYGGGRBGCASCAG-3′ and 5′-GGACTACNNGGGTATCTAAT-3′), respectively. The Illumina NovaSeq platform was used to generate 250-bp paired-end reads. The sequencing was performed on the QIIME2 platform of Beijing Novogene Biotech Co., Ltd. (Beijing, China) and analyzed based on amplicon sequence variants (ASVs) (Li et al., 2020). QIIME2 was used for the analysis of alpha diversity (including Chao1, Observed_otus, Shannon, and Simpson) and principal component analysis (PCA). The dominant microbiota of all groups was detected using LEfSe analysis.

### 2.11 Statistical analysis

The data were analyzed using GraphPad Prism 8 (GraphPad Inc., La Jolla, CA) and presented as mean ± SEM. A one-way ANOVA was used to evaluate statistical differences among experimental groups. Experiments were performed with at least three independent biological replicates, and a *p*-value of < 0.05 was considered statistically significant.

## 3 Results

### 3.1 PINX has no effect on pig weight gain, organ coefficient, and serum biochemical indexes

**Figure 1:**
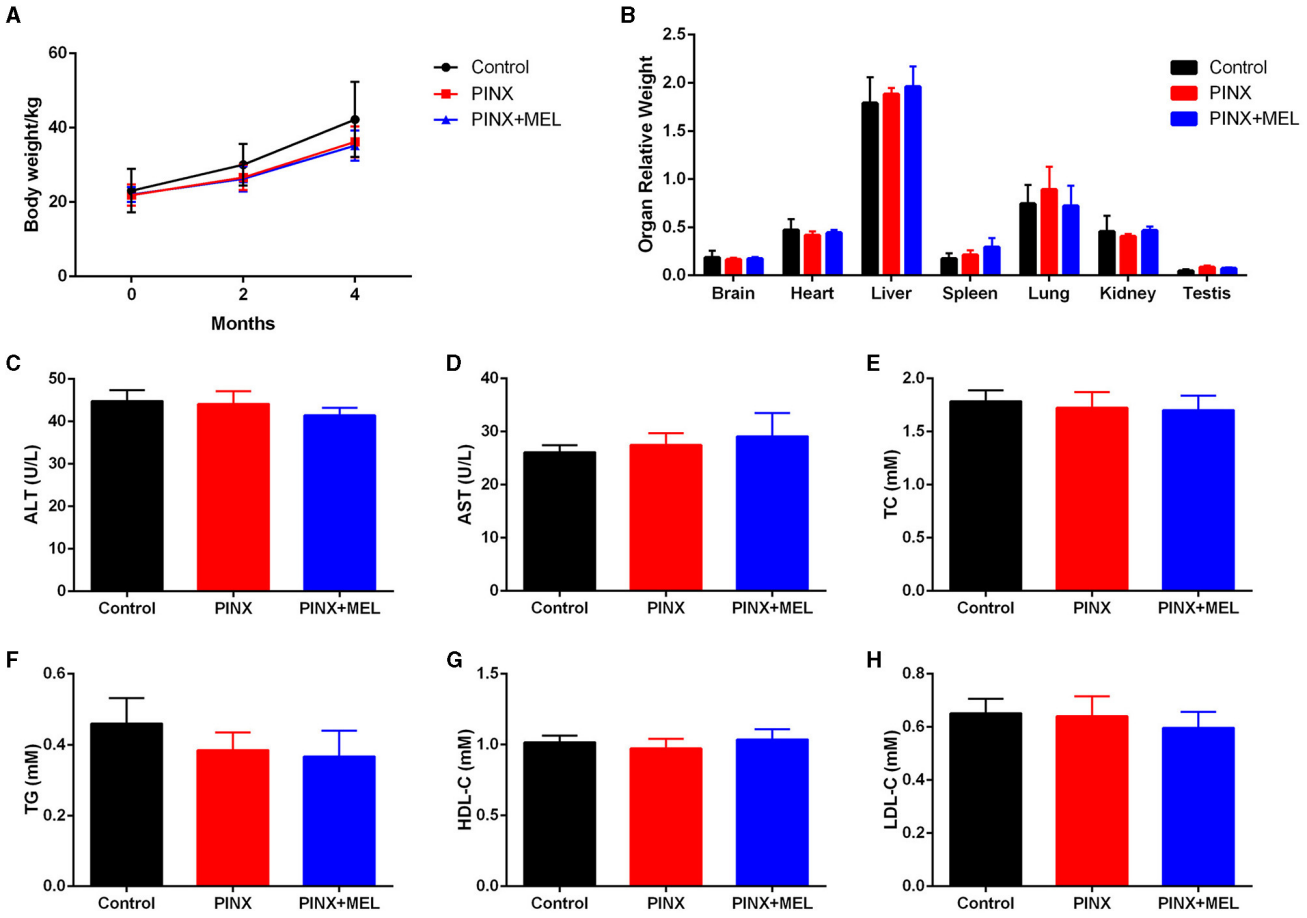
Effects of PINX on body weight, organ coefficients, and serum biochemical indexes in pigs. **(A)** The body weights of three groups of pigs were recorded. **(B)** Effect of PINX on the relative organ weights of the brain, heart, liver, spleen, lungs, kidneys, and testes after 4 months. The effect of PINX on serum levels of ALT **(C)**, AST **(D)**, total cholesterol (TC) **(E)**, triglycerides (TG) **(F)**, high-density lipoprotein cholesterol (HDL-C) **(G)**, and low-density lipoprotein cholesterol (LDL-C) **(H)**. Control, sham–pinealectomy group; PINX, pinealectomy group; PINX+MEL, pinealectomy + melatonin supplementation group.

To confirm the successful establishment of the pig PINX model, the MEL concentration in serum was first assayed. The results showed that PINX dramatically decreased the global MEL level and omitted the diurnal pattern compared with the control (unpublished data) but did not affect the body weight gain (Figure 1A). In addition, PINX and MEL supplementation had no significant effects on the indexes of brain, heart, liver, spleen, lung, kidney, and testis (Figure 1B) and the serum levels of ALT, AST, TC, TG, HDL-C, and LDL-C (Figures 1C–H). These findings suggest that PINX and MEL supplementation do not affect the growth performance in pigs.

### 3.2 PINX does not affect MEL levels in GIT and intestinal contents

**Figure 2:**
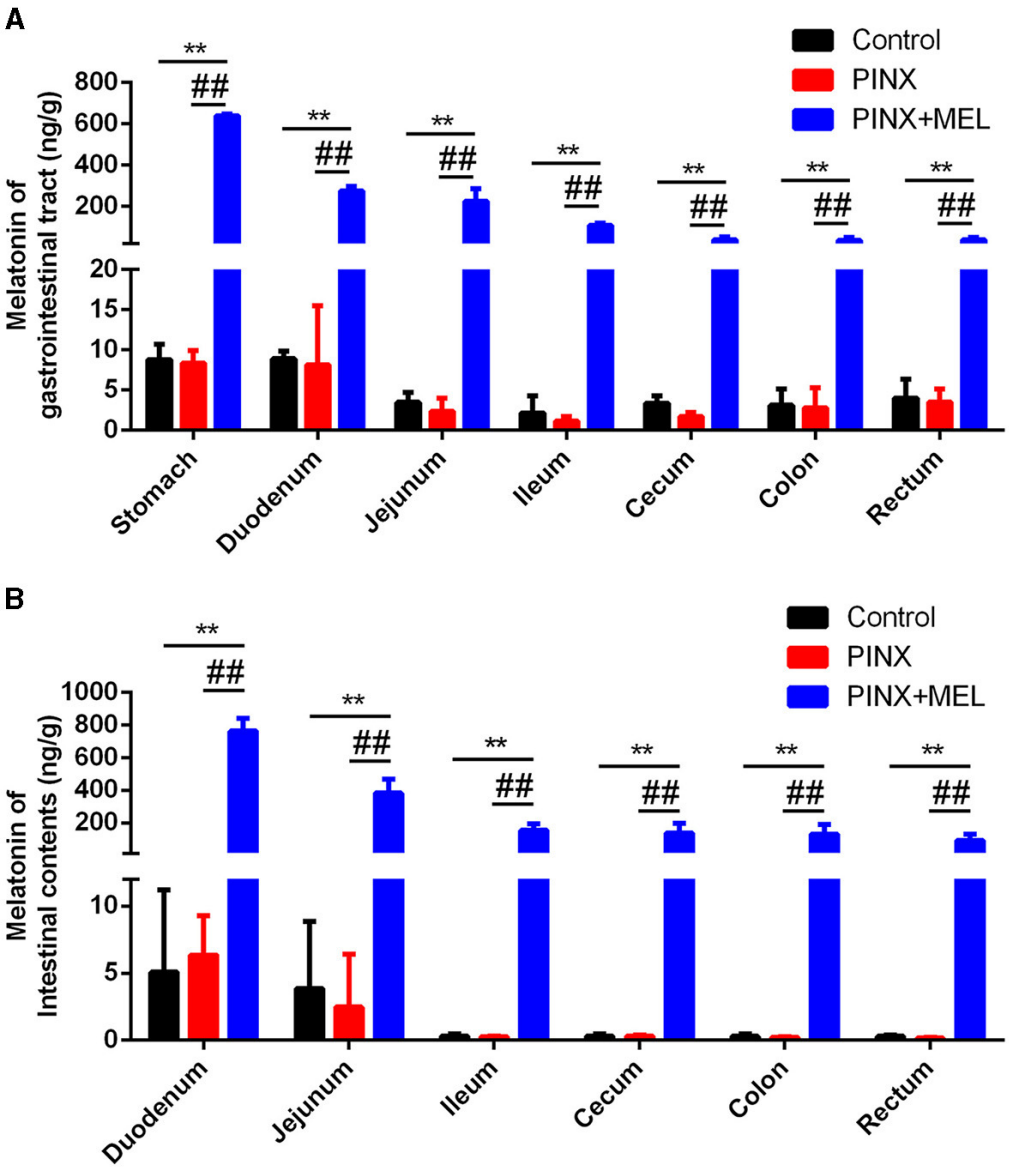
Effect of PINX on MEL concentration in the gastrointestinal tract and intestinal contents. **(A)** MEL levels in different segments of gastrointestinal tract tissues of three groups of pigs. **(B)** MEL levels in different segments of intestinal contents of three groups of pigs. Data are represented as mean ± SEM. **p < 0.01 compared with the control group, ^##^*p* < 0.01 compared with the PINX group.

**Figure 3:**
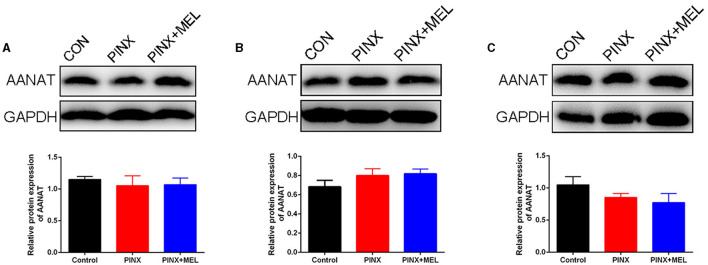
Effect of PINX on AANAT expression in the gastrointestinal tract. Immunoblots (up) and quantification (down) of AANAT in the duodenum **(A)**, ileum **(B)**, and colon **(C)**.

To explore the effect of PINX on the MEL levels in the GIT, the concentration of MEL in different segments of the GIT and intestinal contents was detected. The results showed that the MEL levels in the stomach and duodenum were higher than those in other segments in the control group, but no significant differences were found among the segments of the PINX pigs compared to the control group. In addition, PINX did not affect the MEL levels in the intestinal contents (Figure 2). Furthermore, the AANAT protein expressions in the duodenum, ileum, and colon had no significant differences among the three groups (Figure 3), indicating that MEL synthesis in the GIT is independent of the pineal gland in pigs.

### 3.3 PINX had little effect on the intestinal morphology and intestinal mucosal barrier

**Figure 4:**
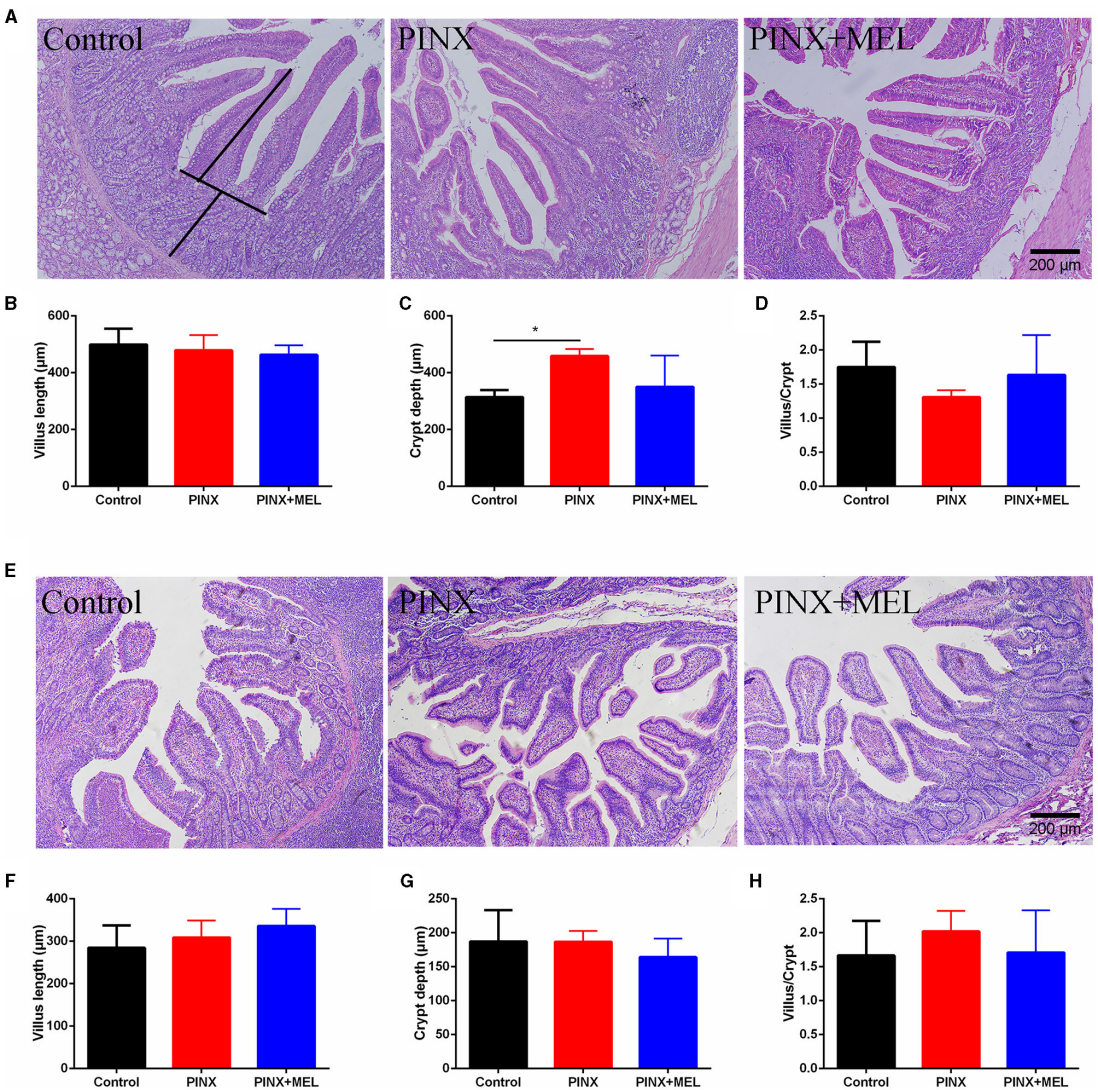
Effect of PINX on duodenal and ileum morphology. **(A)** Representative images of H&E staining in the duodenum. The villus length and crypt depth were measured as indicated in the image. **(B–D)** Statistical analysis of villus length, crypt depth, and the ratio of the villus to crypt of the duodenum. **(E)** Representative images of H&E staining in the ileum. **(F–H)** The statistical analysis of villus length, crypt depth, and the ratio of the villus to crypt of the ileum. Scale bar: 200 μm. Data are represented as mean ± SEM. *p < 0.05 compared with the control group.

**Figure 5:**
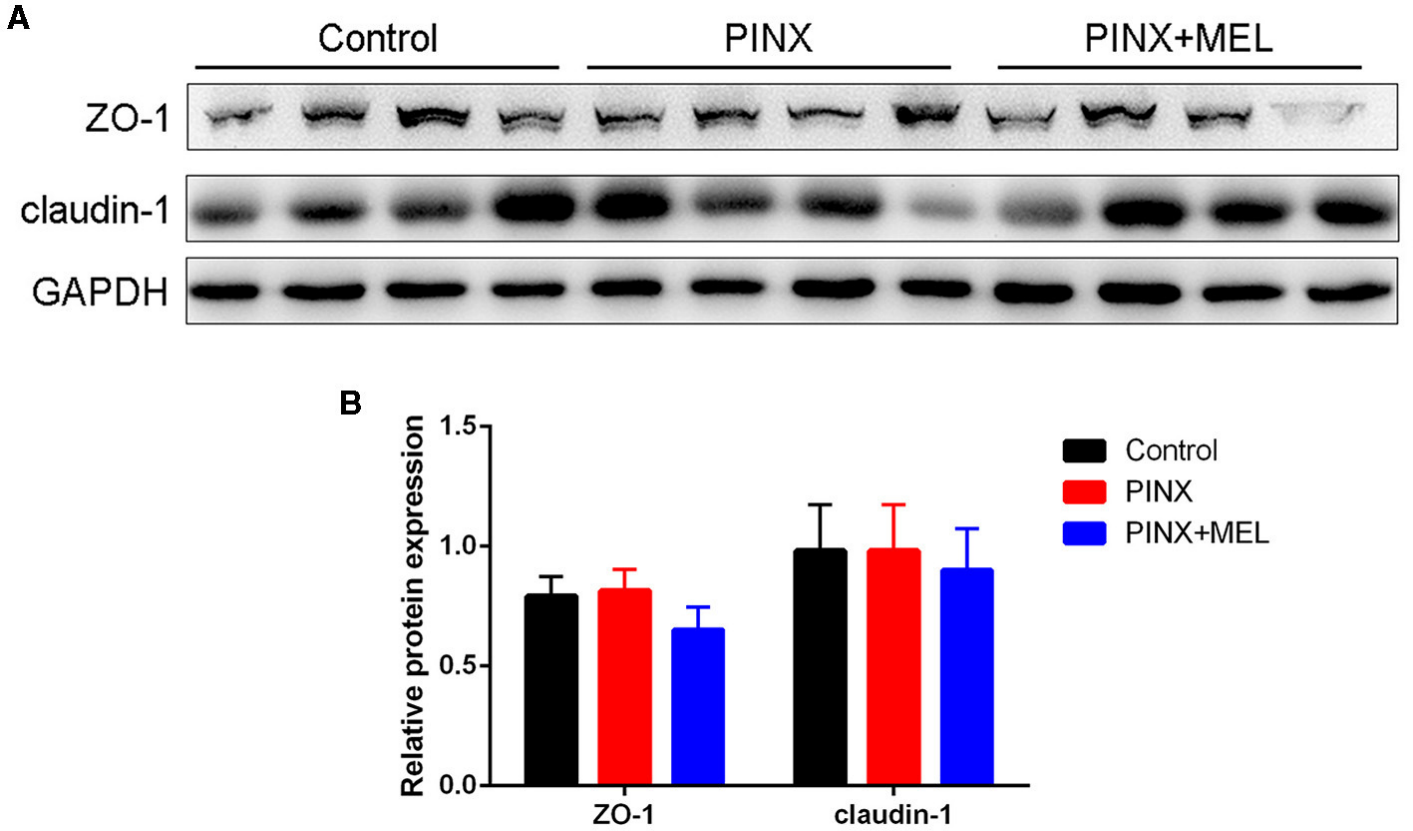
Effect of PINX on the duodenal mucosal barrier. Immunoblots **(A)** and quantification **(B)** of ZO-1 and claudin-1 in the duodenum.

The histological examination showed that PINX and MEL supplementation did not affect the villus height and villus/crypt ratio in the duodenum and ileum, although PINX increased the duodenal crypt depth (Figure 4). Therefore, we explored the cellular mucosal barrier of the duodenum in different groups of pigs. The Western blotting assay revealed that no difference was observed in the protein expression of ZO-1 and claudin-1 among the three groups (Figure 5), suggesting that PINX and MEL supplementation had little effect on duodenal and ileal mucosal morphology and on the mucosal barrier.

### 3.4 Effects of PINX on intestinal microbiota

**Figure 6:**
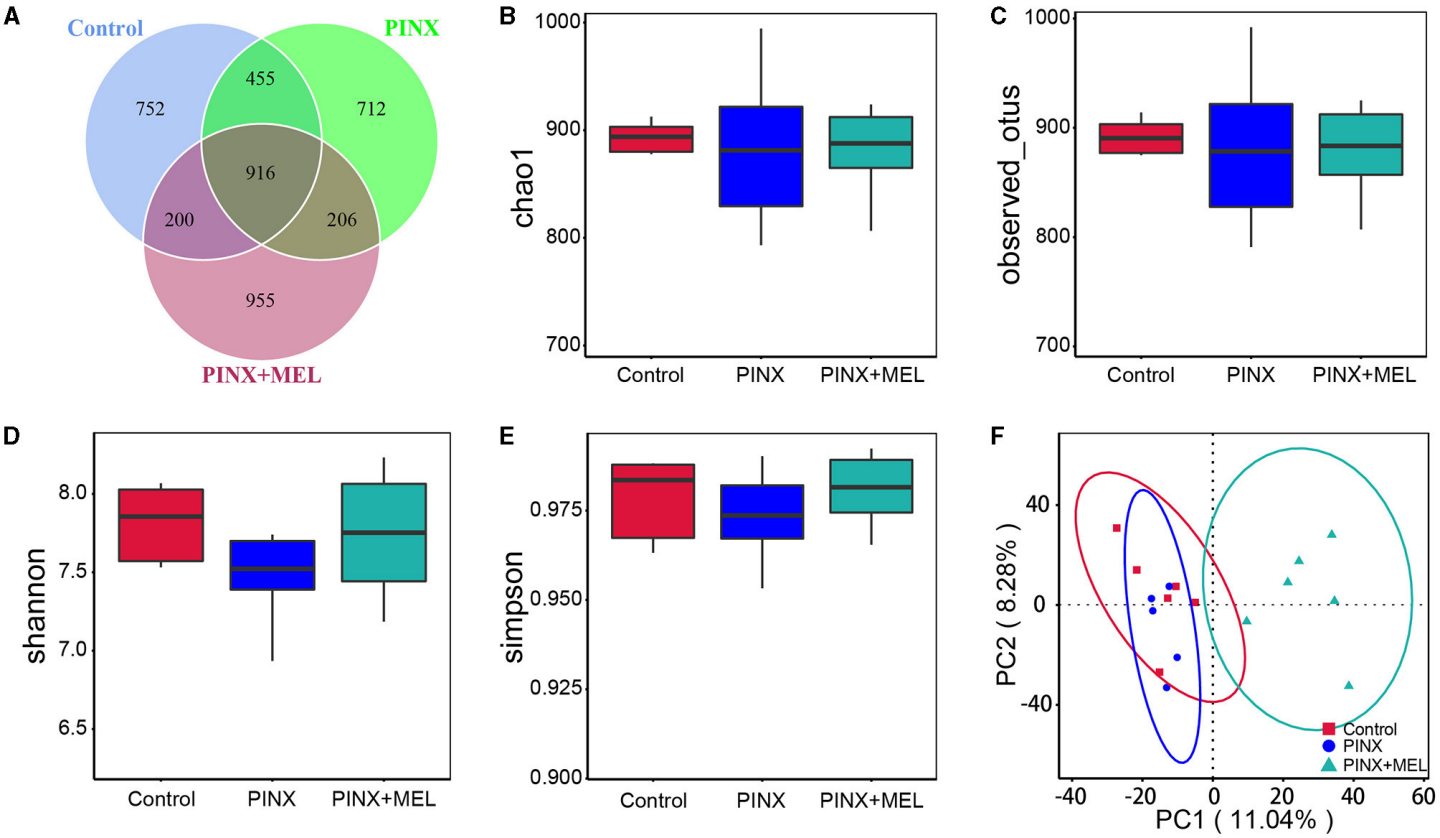
Effect of PINX on colonic microbiota alpha diversity and beta diversity. **(A)** Venn diagram showing overlapping ASVs identified in pig's microbiota of each group. Alpha diversity analysis of Chao1 **(B)**, Observed_otus **(C)**, Shannon **(D)**, and Simpson **(E)**. **(F)** Principal component analysis (PCA) of colonic microbiota.

The Venn diagram showed common and unique ASVs among the treatment groups. A total of 4,196 ASVs were determined, and there were 916 common ASVs among the three groups. In addition, 752, 712, and 955 unique ASVs were specifically identified in the control, PINX, and PINX+MEL groups, respectively (Figure 6A). We further determined four indexes (Chao1, Observed_otus, Shannon, and Simpson) to evaluate the alpha diversity of the microbiome in each group, but no significant differences were observed in the intestinal microbiota α diversity index among the three groups, indicating that PINX and MEL supplementation did not have a significant effect on the richness and diversity of the colonic microbiota (Figures 6B–E). The PCA results showed that the distance between the control and the PINX sample was close, indicating their similarities in the microorganisms. However, the PINX+MEL group showed a distinctive separation from control and PINX treatments (Figure 6F), suggesting that the MEL supplementation changed the microbiota composition of pigs.

**Figure 7:**
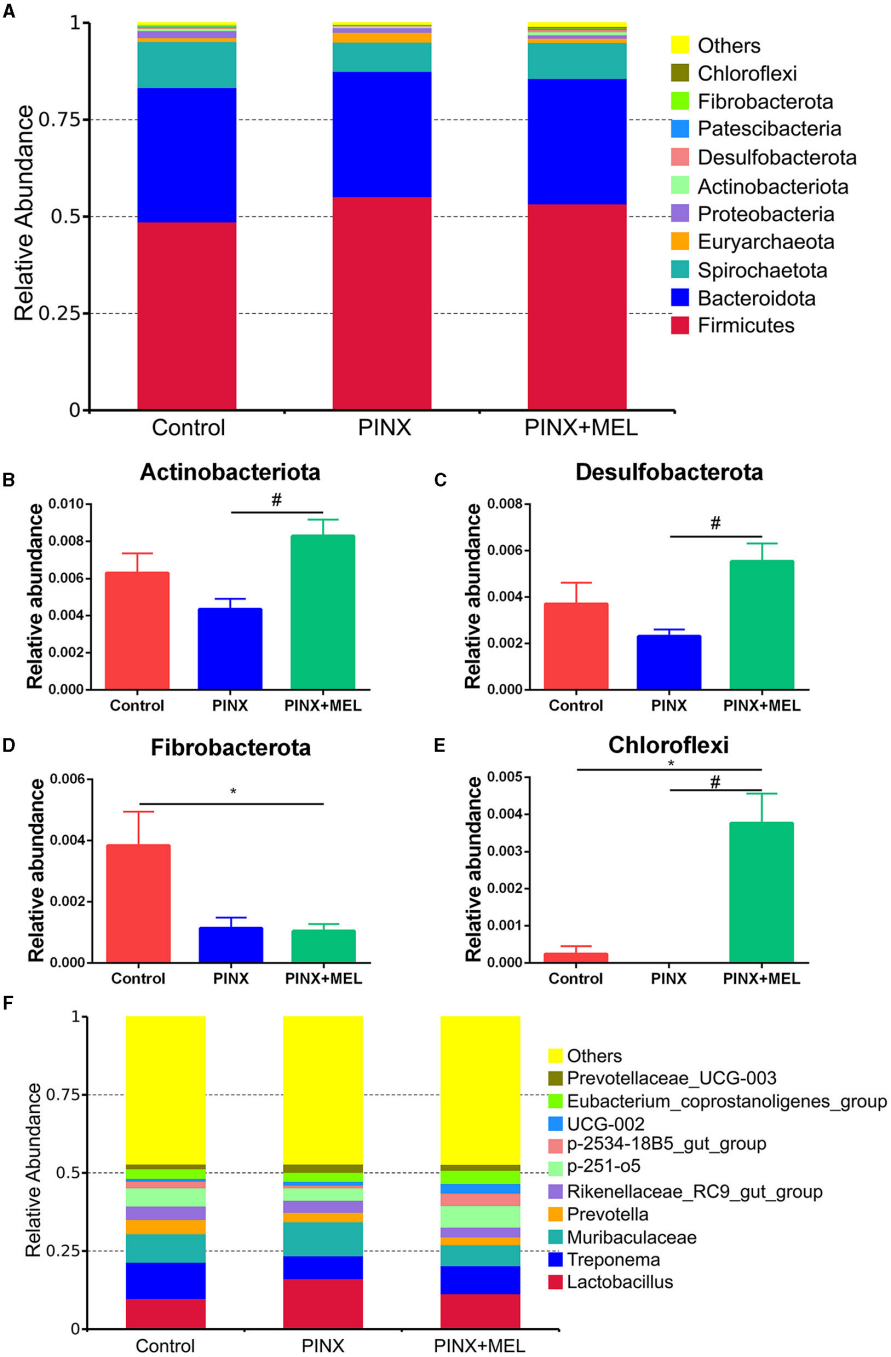
Effects of PINX on gut microbiome composition. **(A)** Relative contribution of the top 10 phyla in each group. **(B–E)** Comparison of the relative abundance at the phylum levels among each group. **(F)** Relative contribution of the top 10 genera in each group. Data are represented as mean ± SEM. *p < 0.05 compared with the control group, ^#^*p* < 0.05 compared with the PINX group.

**Figure 8:**
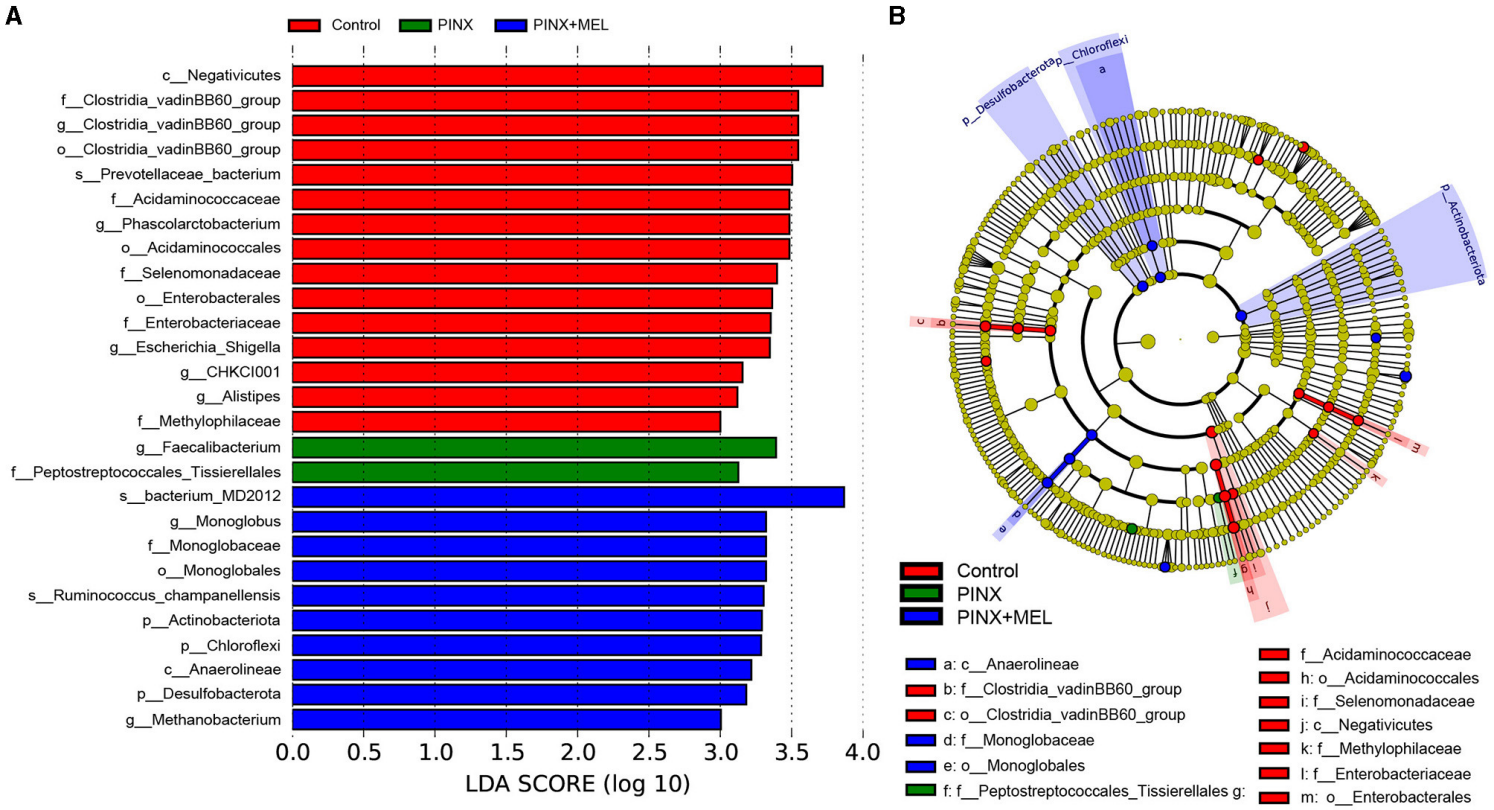
Different taxa microbe analysis based on the LEfSe method. **(A)** Linear discriminant analysis (LDA) score in each group (LDA ≥ 3.0). **(B)** Cladogram of gut microbiota in each group. Each circle's diameter reflects the abundance of that taxa in the community.

The relative abundance of microbiota is given in Figure 7. The phylum level analysis showed that Firmicutes, Bacteroidota, Spirochaetota, Euryarchaeota, Proteobacteria, and Actinobacteriota were the top six phyla. Compared with the control, although PINX decreased the abundance of Actinobacteriota, Desulfobacterota, Fibrobacterota, and Chloroflexi at the phylum level, it was not significant. However, compared to the PINX group, MEL treatment decreased Fibrobacterota abundance and increased Actinobacteriota, Desulfobacterota, and Chloroflexi abundance. At the genus level, PINX and MEL supplementation had no effect on the relative abundances of microbiota. To identify the distinct gut microbiota of the three groups, we conducted the linear discriminant analysis (LDA) effect size (LEfSe). The results showed that Negativicutes, Clostridia_vadinBB60, Prevotellaceae_bacterium, Acidaminococcaceae, and *Phascolarctobacterium* were significantly more abundant in the control group. In addition, Faecalibacterium and Peptostreptococcales_Tissierellales are domainant in PINX pigs. MEL supplementation significantly elevated the relative abundance of bacterium_MD2012, *Monoglobus* (Monoglobaceae, Monoglobales), Ruminococcus_champanellensis, Actinobac-teriota, Chloroflexi, Anaerolineae, Desulfobacterota, and *Methanobacterium* (Figure 8). Collectively, these results demonstrated that PINX does not affect the gut microbiota in pigs, but MEL supplementation changed the composition of the gut microbiota.

## 4 Discussion

It has been documented that MEL supplementation affects the gut microbiota composition in mice (Xu P. et al., 2017), rat (Lv et al., 2020), and suckling piglets (Xia et al., 2022). In this study, the effect of endogenous MEL deficiency on gut microbiota has been studied using a new established pig PINX model. The results demonstrate that PINX has no significant effect on MEL concentration in the intestine and intestinal contents, as well as on the intestinal microbiota, suggesting that MEL synthesis in the GIT is independent of the pineal gland, supported by the findings of Bubenik and Brown (1997) that PINX has no influence on MEL levels in the intestine. Meanwhile, MEL supplementation has considerable effect on shaping the composition of the intestinal microbiota.

In the current study, we observed that PINX significantly reduced nighttime serum MEL levels, confirming the successful removal of the pineal gland. However, GIT enterochromaffin cells were reported to synthesize MEL and contribute to plasmatic MEL concentration (Huether et al., 1992; Bubenik and Brown, 1997), so the serum MEL is detectable even after PINX. Studies have shown that MEL has anti-obesity effects, reducing TG and TC concentrations and liver steatosis in mice fed with a high-fat diet (Xu P. et al., 2017). However, the current study found that PINX and MEL supplementation had no effect on body weight gain and serum biochemical indexes of pigs fed with basic diets, which was similar to the results observed in PINX rats (Buonfiglio et al., 2018) and MEL-supplemented mice (Haridas et al., 2013) and piglets (Xia et al., 2022). Possible explanations for this difference may include the animal species, experimental duration, and feeding regimes. In our study, it should be noted that the pigs were fed with a basic diet according to their weight, while the mice or rats had *ad libitum* access to food in other studies. These results indicate that PINX and MEL supplementation has no effect on growth performance in pigs.

The mucosal morphology parameters, such as villus height, crypt depth, and tight junction protein, are often used to evaluate intestinal barrier function (Ding and Li, 2003). Ren et al. (2018) found that MEL increased the ratio of the villus to crypt in the ileum, promoted nutrient absorption, and consequently improved body weight gain in mice with weanling stress. Gao et al. (2019) found that sleep deprivation damaged the tight junction proteins, resulting in a loss of intestinal mucosal integrity. In this study, although PINX decreased the MEL levels in serum and sleep time in pigs during the dark phases (data not shown), PINX did not influence the intestinal integrity, as reflected by the levels of proteins ZO-1 and claudin-1. These inconsistent results may be due to the differences in the duration of the experiment. Gao et al. conducted an acute sleep deprivation experiment with a duration of 3 days, while in our study, the pineal gland was removed for 4 months.

Recently, an interesting study showed a new role of the pineal gland in regulating seasonal alterations of gut microbiota in Siberian hamsters (Shor et al., 2020). In this study, we demonstrated that PINX had no significant effects on the alpha diversity and beta diversity of the intestinal microbiota, whereas the beta diversity analysis showed that MEL supplementation altered the microbial community structure. It is acknowledged that the Firmicutes-to-Bacteroidetes ratio is considered to be an important indicator for the state of the intestinal microbiota, and previous studies have reported that MEL affects the Firmicutes-to-Bacteroidetes ratio (Xu P. et al., 2017; Yin et al., 2018, 2020). In this study, the abundance of Firmicutes and Bacteroidetes was not significantly different after MEL supplementation; this finding is consistent with previous results in suckling piglets (Xia et al., 2022). This discrepancy may be caused by differences in model organisms (mice versus pigs). However, Ruminococcaceae, a beneficial bacterium belonging to the phylum Firmicutes, may ameliorate intestinal inflammation (Wang L. et al., 2021; Wang Y. et al., 2021). Butyrate, a primary product of intestinal microbial fermentation of dietary fiber, serves as an essential energy source for intestinal epithelial cells and protects colonic epithelial cells from tumorigenesis (Hamer et al., 2008; Xu S. et al., 2017; Yang and Yu, 2018). Ruminococcaceae is a major producer of butyrate, which is dramatically reduced in individuals suffering from inflammatory bowel disease (Darnaud et al., 2018; Ma et al., 2023). A previous study revealed that MEL effectively ameliorated sleep deprivation-induced colitis by restoring intestinal microbiota homeostasis and improving the production of butyrate (Gao et al., 2021). In this study, LEfSe analysis showed that *Ruminococcus* was much more abundant in the MEL supplementation group. The increase in the abundance of *Ruminococcus* may increase the production of butyrate, which contributes to a healthier intestinal environment. In addition, MEL supplementation decreased Fibrobacterota abundance and increased the relative abundance of Actinobacteriota, Desulfobacterota, and Chloroflexi. Fibrobacterota is important for the degradation of cellulose in the gastrointestinal tracts, best known for its role in rumen function, and is a potential source of novel enzymes for bioenergy applications (Jewell et al., 2013). It is believed that cellulose hydrolysis, anerobic metabolism, and the absence of motility are the unified characteristics of Fibrobacterota (Abdul Rahman et al., 2015). A recent study suggests that indole-3-carboxaldehyde, a tryptophan metabolite produced by bacteria, decreased the abundance of phylum Fibrobacterota, which had a beneficial role in intestinal health in weaned piglets (Zhang et al., 2022). Thus, we speculated that MEL, which is also a tryptophan derivative and an indole-like neuroendocrine hormone, may have the same inhibitory mechanism on Fibrobacterota and improve intestinal health. Another bacteria phylum, Actinobacteria, is known to generate various antimicrobial compounds and maintain mucosal immunity (Arango et al., 2016), which have a variety of anti-tumor and anti-biofilm properties (Azman et al., 2019). It is estimated that over 80% of medicinal antibiotics are derived from Actinobacteria, especially the *Streptomyces* genus (Procópio et al., 2012). Importantly, butyrate can ameliorate the progression of colorectal cancer induced by AOM/DSS in mice, partially by promoting the colonization of Actinobacteriota (Kang et al., 2023). Our findings are supported by those of a recent study on piglets, which revealed that MEL treatment enhanced the proportion of Actinobacteria, potentially augmenting aspects such as barrier integrity, nutrient absorption, and microbial balance (Xia et al., 2022). Members of the Desulfobacterota phylum exhibit a predilection for anoxic environments, with many using sulfur compounds or iron as the terminal electron acceptors during respiration and/or disproportionation reactions (Simon and Kroneck, 2013; Murphy et al., 2021). This process is essential for the host's energy metabolism, as sulfate is a key source of sulfur for protein synthesis and other cellular processes. It is worth noting that rice bran diet improved intestinal digestive enzyme activities, increased Desulfobacterota abundance, and promoted growth in pigs (Li et al., 2023). In addition, polysaccharides can increase the relative abundance of Verrucomicrobiota, Desulfobacterota, and Actinobacteriota; generate short-chain fatty acids; and benefit intestinal health (Hu et al., 2021). While the role of Chloroflexi in the mammalian gut is less understood, the increased abundance of Chloroflexi following MEL supplementation may suggest its potential role in shaping the gut environment or metabolite profile. Collectively, our study sheds light on the multifaceted impact of MEL supplementation on the gut microbiome. These shifts in microbial communities likely contribute to an overall enhancement of gut health and function.

In summary, to the best of our knowledge, this is the first study to investigate the effects of PINX on GIT MEL levels and the composition of intestinal microbiota in pigs. The results support that MEL synthesis and gut microbiota homeostasis in the GIT are independent of the pineal gland in pigs. MEL supplementation modulates the composition of intestinal flora. However, future research studies are needed to elucidate the molecular mechanism by which MEL participates in host–bacteria interactions.

## Data availability statement

The datasets presented in this study can be found in the NCBI SRA Repository (https://www.ncbi.nlm.nih.gov/sra), accession number PRJNA1086051.

## Ethics statement

The animal study was approved by Animal Ethics Association of Yangzhou University [approval ID: SYXK (Su) 2022-0044]. The study was conducted in accordance with the local legislation and institutional requirements.

## Author contributions

JZ: Writing – original draft, Project administration, Methodology, Data curation. YZ: Writing – original draft, Project administration, Methodology. DZ: Writing – review & editing, Project administration, Methodology. KM: Writing – review & editing, Project administration, Methodology. YG: Writing – review & editing, Project administration, Methodology. XL: Writing – review & editing, Project administration, Methodology. JL: Methodology, Writing – review & editing, Supervision, Funding acquisition. SC: Methodology, Writing – review & editing, Supervision, Funding acquisition.
